# MicroRNAs in Pancreatic Cancer: Advances in Biomarker Discovery and Therapeutic Implications

**DOI:** 10.3390/ijms25073914

**Published:** 2024-03-31

**Authors:** Roland Madadjim, Thuy An, Juan Cui

**Affiliations:** School of Computing, University of Nebraska—Lincoln, Lincoln, NE 68588, USA; rmadadjim2@huskers.unl.edu (R.M.); thuy.an@huskers.unl.edu (T.A.)

**Keywords:** pancreatic cancer, microRNAs, exosomal microRNAs, biomarkers, diagnosis, prognosis, targeted therapy

## Abstract

Pancreatic cancer remains a formidable malignancy characterized by high mortality rates, primarily attributable to late-stage diagnosis and a dearth of effective therapeutic interventions. The identification of reliable biomarkers holds paramount importance in enhancing early detection, prognostic evaluation, and targeted treatment modalities. Small non-coding RNAs, particularly microRNAs, have emerged as promising candidates for pancreatic cancer biomarkers in recent years. In this review, we delve into the evolving role of cellular and circulating miRNAs, including exosomal miRNAs, in the diagnosis, prognosis, and therapeutic targeting of pancreatic cancer. Drawing upon the latest research advancements in omics data-driven biomarker discovery, we also perform a case study using public datasets and address commonly identified research discrepancies, challenges, and limitations. Lastly, we discuss analytical approaches that integrate multimodal analyses incorporating clinical and molecular features, presenting new insights into identifying robust miRNA-centric biomarkers.

## 1. Introduction

Pancreatic cancer (PC) stands as one of the most lethal malignancies, with a mere 9% five-year survival rate. Typically diagnosed in advanced stages, treatment options remain limited, with only 15–20% of cases amenable to resection upon diagnosis, leading to a dismal prognosis [1]. The critical necessity for early detection of pancreatic cancer is undeniable yet hindered by the absence of specific symptoms and effective screening methodologies. Current diagnostic methods, exemplified by the best-validated PC biomarker, carbohydrate antigen 19-9 (CA19-9), exhibit insufficient sensitivity and specificity for early screening, underscoring the urgent need for innovative approaches [2].

MicroRNAs (miRNAs), a class of small non-coding RNAs, typically 18–25 nucleotides in length, play pivotal roles in post-transcriptional gene regulation by binding to the 3′ untranslated region (UTR) of target messenger RNAs (mRNAs) that induces mRNA degradation or translational repression [3,4,5,6,7]. Beyond their significance in cellular progresses such as growth, immunity, and nutrient homeostasis, dysregulated miRNAs are implicated in cancer development and progression [8,9,10,11,12]. Numerous miRNAs have been identified with oncogenic or tumor-suppressing functions since they may regulate target genes that promote or inhibit tumor growth [13,14]. However, only a few have been fully validated. MiR-7, miR-21, and miR-155 are among the most researched ones; they have been linked to major cancer hallmarks in most solid tumors, including pancreatic cancer, such as enhanced cell proliferation, migration, and invasiveness, re-induced apoptosis, and immune evasion [15,16,17,18,19,20].

It is well noted that miRNAs may act as oncogenes, named oncomiRs, in some types of cancers but tumor suppressors in others, depending on their specific expressions and the cellular context. In pancreatic cancer, in addition to those reviewed in [21,22], newly reported tumor suppressor miRNAs include miR-634 [23], miR-1225 [24], miR-623 [25], miR-345-5p [26], and miR-30c [27], while oncomiRs include miR-194 [28] and miR-221 [29]. Their altered expression patterns in pancreatic cancer versus controls make them promising diagnostic and prognostic indicators [9,10,30,31,32,33]. Despite ongoing efforts [34,35,36,37], the roles of miRNA in the etiology and progression of pancreatic cancer are often not fully studied. This is partially attributed to the lengthy and challenging process of determining miRNA functions, which requires reliable target identification through computational predictions and extensive experimental validations. Given that a single miRNA can simultaneously target multiple genes, while a single gene can be targeted by multiple miRNAs, which also varies in conditions, modeling such complex miRNA–gene interactions should be supported by a sophisticated systematic network analysis [38,39].

The miRNA expression profiles obtained from various biopsy methods offer valuable insights into pancreatic tumor molecular characteristics, serving as a comprehensive pool of potential biomarkers for clinical use. Aberrant miRNA expression patterns in pancreatic cancer tissues and biofluids, analyzed through techniques like PCR, a microarray, and small RNA sequencing (sRNA-seq), hold promise for distinguishing cancer patients from healthy individuals [9,33,40,41]. For instance, elevated expressions of tissue miR-21, miR-155, and miR-451 have demonstrated promising diagnostic value in pancreatic cancer [20,42].

Blood miRNAs, due to their stability and minimally invasive sampling, are increasingly recognized as robust biomarkers for diagnosis, prognosis, and monitoring of pancreatic cancer [43]. Serum miR-21, for example, was used as a predictor for chemosensitivity of advanced pancreatic cancer [44]. Exosomal miRNAs, encapsulated within extracellular vesicles (EVs), present themselves as promising candidates for biomarker discovery, owing to their implication in pancreatic carcinogenesis, stability, and non-invasive detection potential [40,45,46,47,48,49,50,51].

In contrast to the single miRNA biomarkers, combining multiple miRNAs or other molecules (e.g., CA19-9) as signatures can enhance the accuracy of pancreatic cancer diagnosis and prognosis [50,52,53,54,55,56,57,58,59,60,61]. Analytical approaches have evolved from simple statistical tests identifying significant individual miRNAs differentially expressed in cancer versus control, e.g., Student’s *t*-test and ANOVA, to sophisticated computational methods, such as Linear and Quadratic Discriminant Analysis, LASSO regression, and machine learning [62,63,64,65], integrating miRNA profiles with other clinical and molecular markers to enhance predictive power [8,66].

To provide an overview of the most recent research progress on miRNA biomarkers in pancreatic cancer, we screened the titles and abstracts of over 900 articles published in PubMed between 2014 and 2024 that include the keywords “microRNA, biomarker, and pancreatic cancer”. We focus on miRNAs identified in human pancreatic tissues and biofluids with diagnostic and prognostic potential by large omics-based studies or with therapeutic implications verified by both in vitro and in vivo experiments. In addition, we conducted a case study utilizing public miRNA expression datasets collected from the Gene Expression Omnibus (GEO) repository (https://www.ncbi.nlm.nih.gov/geo/, 1 August 2023). The discussion focuses on significant achievements, lessons learned, potential limitations and challenges, and possible solutions by identifying reliable combinatory biomarkers. Specifically, with a focus on circulating exosomal miRNAs, the aim is to elucidate their potential for non-invasive liquid biopsy in early PC screening alongside new approaches focusing on multimodal analysis and mechanistic discovery.

## 2. MiRNAs Biomarkers in Pancreatic Cancer

### 2.1. Diagnostic Potential of miRNAs in Pancreatic Cancers

The potential of aberrant miRNA expression for cancer diagnosis and prognosis has been widely recognized, prompting comparative analyses of miRNA abundances measured by various techniques to identify potential markers. Numerous prior studies have reported an aberrant expression of known cancer-related miRNAs in pancreatic ductal adenocarcinoma (PDAC) tissues, including a downregulation of tumor suppressor miRNAs such as miR-15a, miR-16-1, miR-126, miR-200, and miR-let-7, alongside the upregulation of oncogenic miRNAs such as miR-21, miR-221, and miR-155, as comprehensively reviewed by Kabiraj and Kundu [12]. More recently, tissue miR-483-3p [41], miR-1307 [11], and several others [42,67] have been found to be dysregulated (Table 1), further emphasizing the diagnostic potential of these miRNAs. In these studies, the diagnostic power of the presented biomarkers is either supported by the AUC (Area Under Curve) measure or revealed implicitly by the statistical significance level of their differential expressions.

Contrary to tissue miRNAs, those circulating in blood and other body fluids offer a potential pool of non-invasive biomarker candidates [83]. Studies by Kojima et al. [31] and Kim et al. [68], using small RNA sequencing on serum samples from pancreatic cancer, biliary tract cancer (BTC), and control groups revealed differentially expressed miRNAs, with subsequent validation studies identifying promising candidates such as miR-744-5p, miR-409-3p, and miR-128-3p. Khan et al. identified a few serum miRNAs (miR-320b, miR-215-5p, miR-192-5p, miR-122-5p, miR-30b-5p, and others) with significant accuracy (AUC: 0.720–0.988), further highlighting the potential of circulating miRNAs in diagnostic applications [67]. Additionally, Hussein et al. identified the plasma miRNAs miR-22-3p, miR-642b-3p, and miR-885-5p, which were significantly upregulated in pancreatic cancer, indicating high diagnostic accuracy and corroborating the diagnostic potential of miR-642b-3p identified in prior research [69]. Other recently identified markers include serum miR-25 [10,70,71]; plasma miR-34a-5p, miR-130a-3p, and miR-222–3p [72]; and blood/urine miR-1246 [73], which warrant further tests on larger patient groups.

Complementing single gene biomarkers, studies focusing on combinatory signature detection have shown promising results. For instance, a 39-miRNA signature was identified through a machine learning model that can differentiate PC versus controls with an accuracy of 0.93 and AUC of 0.98 [59]. Shams et al. performed an integrative bioinformatics analysis, identifying 27 differentially expressed serum miRNAs with diagnostic potential (AUC > 0.8) and proposing several miRNA panels with varying accuracies. The most promising signature consisting of mir-125a-3p, mir-5100, and mir-642b-3p shows an AUC of 0.95, sensitivity of 0.98, and specificity of 0.97, outperforming other similar panels [74]. Similarly, a recent study reported a compelling signature comprising four serum miRNAs (miR-132-3p, miR-30c-5p, miR-24-3p, miR-23a-3p) for PC detection with promising accuracy (AUC (0.971)), based on the analysis of tissue and serum samples from 1273 participants [75]. In addition, a panel of 2′-O-methylated (2′OMe) miRNAs (miR-28-3p, miR-143-3p, miR-151a-3p) was found upregulated in the PDAC versus control with the validation AUC of 0.928 [76]. Another similar plasma panel, including miR-125a-3p, miR-4530, and miR-92a-2-5p, shows a slightly lower performance with an AUC of 0.86 [77].

Next, circulating exosomal miRNAs have emerged as promising non-invasive biomarkers for early detection. In a recent review article [12], Kabiraj and Kundu corroborated the significance of circulating exosomal miRNAs, specifically highlighting the dysregulation of miR-21, miR-155, miR-196a, miR-200b, and miR-200c in tumorigenesis and their diagnostic and prognostic merit in multiple cancer types. In pancreatic cancer, recent studies have identified exosomal miR-196a and miR-1246 from plasma [78]; exosomal miR-191, miR-21, miR-451a, and others from serum [52,64]; EV-derived miR-18a, miR-106a, and miR-664a-3p from plasma [79,80]; miR-3940-5p and miR-8069 from urine [81]; miR-1246 and miR4644 from saliva [82]; and EV-derived miR-20a from duodenal fluid [57], showing diagnostic potential in PDAC, pancreatobiliary tract cancer (PTC), and intraductal papillary mucinous neoplasms (IPMN), respectively. Similarly, Lai et al. have identified an elevated expression of serum miR-21, miR-30c, miR-106b, miR-20a, miR-181a, and miR-10b and suppressed expression of miR-483 and miR-122, which can differentiate PDAC from chronic pancreatitis (CP) [52]. Another pilot study has indicated that miR-20a from duodenal-fluid EVs can be a potential biomarker for PDAC detection [57].

Furthermore, incorporating miRNA with other circulating markers has further improved diagnostic sensitivity [40]. For instance, Nakamura et al. demonstrated an enhanced diagnostic accuracy, from 74–89% to 91%, when combining pancreatic juice-derived exosomal miRNAs, miR-21, and miR-155 with pancreatic juice cytology (PJC) for differentiating PDAC from CP patients [60]. Combining serum miR-21-5p with human satellite II (HSATII) RNA level can also improve the diagnostic performance (with an AUC of 0.90 compared to 0.87) by using one marker alone [56].

Overall, current evidence underscores the potential of exosomal miRNAs as non-invasive, high-sensitivity biomarkers for the early detection and monitoring of pancreatic cancer.

### 2.2. Prognostic Potential of miRNAs in Pancreatic Cancer

The expression levels of specific miRNAs have been found to correlate with cancer progression, treatment response, and patient survival in pancreatic cancers. Numerous studies have identified miRNA signatures associated with the disease, facilitating the stratification of cancer patients. As summarized in Table 2, elevated levels of serum miR-451a [47], plasma miR-221-3p [29], exosomal miR-21 [64], and cyst fluid EV-derived miR-200b were found in pancreatic cancer patients compared to healthy controls. The increased expression levels of these miRNAs are linked to poorer outcomes, recurrences, and advanced disease stages. For instance, an elevated expression of miR-221-3p was found in PC tissues, cell lines, and plasma, and the expression level of plasma miR-221-3p was correlated with distant metastasis and TNM stages [29]. Similarly, an upregulation of plasma miR-370-3p expression correlates with poor prognosis in PDAC patients (hazard ratios (HR) 2.13, *p* = 0.004) [84]. The overexpression of tissue miR-31 and miR-205 was significantly correlated with higher histological grades and decreased survival in TCGA PDAC clinical samples [85]. In another study [86], miR-200b is present at a higher level in EVs from mucinous pancreatic cystic neoplasms’ (M-PCNs) EVs compared to EVs isolated from other pancreatic cysts (not-M samples). Its expression discriminates patients with these two groups with a higher sensitivity (83.3%) and specificity (90.9%) than other diagnostic methods.

Conversely, decreased levels of tissue miR-132 [30], miR-7 [32], and miR-26a-5p [87] have been observed in pancreatic cancer patients compared to healthy controls. The expression of these miRNAs is associated with tumor progression and metastasis. In [30], it shows that the 1-year survival rate of patients with high miR-132 expression, a tumor suppressor, was greater than that of patients with low miR-132 expression, and the lower expression was associated with poorer prognosis in PDAC. Similarly, low miR-7 expression may contribute to tumor progression and poor prognosis in pancreatic cancer [32]. The elevated expression of miR-26a-5p, an oncomiR, was found to inhibit PDAC cell proliferation, migration, and invasion by targeting ARNTL2, which is correlated to a favorable prognosis [87].

Recent research by Chen et al. analyzed public datasets from TCGA and GEO and identified four downregulated miRNAs (miR-424, miR-3613, miR-4772, miR-126) significantly associated with prognosis in pancreatic cancer [53]. The integration of miRNA target prediction and a network analysis revealed nine key predicted targets (*MMP14*, *ITGA2*, *COL1A1*, *COL3A1*, *COL11A1*, *COL6A3*, *COL12A1*, and *COL5A2*), all upregulated, enriched in pathways like ECM-receptor interaction and focal adhesion. In another study, among all ten miRNAs identified with diagnostic value, several combinations of two miRNAs are significantly associated with short overall survival in PDAC and ampullary adenocarcinomas (A-AC) in combination, e.g., miR-148a and miR-212-3p with HR = 1.20 (95% confidence intervals: 1.09–1.33) [42]. Similarly, a signature comprising miR-574-5p, miR-1244, miR-145, miR-328, miR-26b, and miR-4321 has been reported as a prognostic model for pancreatic cancer, particularly associated with the immune environment [54]. Furthermore, a few circular RNAs were identified with prognostic potential through sponging respective miRNAs, such as circ-0005105/miR-20a-3p [88], circ_0007367/miR-6820-3p, and targeted human genes involved in pancreatic cancer development [80].

### 2.3. Therapeutic Implications of miRNAs in Pancreatic Cancers

In addition to their diagnostic and prognostic roles, miRNAs show promise for targeted therapies in pancreatic cancer. For example, the upregulation of miR-103 in PDAC leads to increased tumor metastasis and a poor prognosis through regulating target *USP10* (ubiquitin-specific peptidase 10) [90]. MiRNA-based therapies involve the restoration or inhibition of specific miRNAs to modulate oncogenic or tumor-suppressive pathways. Clinical trials evaluating miRNA-targeted therapies are underway, providing a novel avenue for personalized treatment.

In a review by Tesfaye et al. [22], the aberrant expression of miRNAs and their target genes and pathways in PDAC, such as oncogene *KRAS* and tumor suppressor genes like *p53*, *p16*, and *SMAD4*, were examined. Key observations included the loss of tumor suppressive miRNAs leading to an overexpression of oncogenes (let-7, miR-34, and miR-200), miRNAs conferring resistance to chemotherapy (miR-21 and miR-221/222), and the sensitization of cells to gemcitabine upon restoration of tumor suppressor miRNAs (miR-34). Similarly, Amartya et al. reviewed the potential therapeutic implication of miRNA with respect to hallmarks in PDAC [21]. In Table 3, we summarize a list of miRNAs dysregulated in PDAC whose therapeutic potential have been verified by both in vitro and in vivo experiments.

Several miRNAs were overexpressed in PDAC samples and cells compared with normal controls, including miR-21, miR-27, miR-194-5p, and miR-29. They play pivotal roles in regulating key signaling and cellular pathways in PDAC. An elevated level of miR-21 was shown to promote drug resistance to 5-FU and enhance the proliferation of pancreatic cancer cells by downregulating the expression of its target genes phosphatase and tensin homolog (*PTEN*) and programmed cell death factor 4 (*PDCD4*) [91]. It also promotes EGF-induced cell proliferation by targeting Sprouty RTK Signaling Antagonist 2 (*Spry2*) [92]. Exosomal miRNA-27a derived from pancreatic cancer cells promotes the angiogenesis of human microvascular endothelial cells in pancreatic cancer via regulating BTG2 [93]. MiR-194-5p downregulates tumor cell PD-L1 expression and promotes anti-tumor immunity in pancreatic cancer [28]. The upregulation of miR-29a was found to be positively correlated with metastasis, and its ectopic expression increased the expression of pro-inflammatory factors, epithelial–mesenchymal transition (EMT) markers, and cell viability and invasion by downregulating Tristetraprolin (*TTP*).

In contrast, the downregulation of a few tumor-suppressing miRNAs was observed in PDAC cells. The ectopic expression of miR-15a was also found to cause cell cycle arrest and inhibit PDAC cell proliferation and EMT via the downregulation of *Bmi-1* expression [94]. MiR-145 can act as a suppressor to slow pancreatic cancer progression by suppressing cancer cell proliferation, migration, and invasion through regulating different targets, including *MUC13*, *the TGF-β receptor*, and *SMAD2* [95,96]. MiR-34a inhibits the migration and invasion of pancreatic cancer cell lines through regulating EMT and Notch signaling via targets *Snail1* and *Notch1* [97].

Epigenetic mechanisms were also noted to regulate miRNA expression [22]. More recently, Zheng et al. demonstrated the oncogenic role of a circular RNA derived from exons of myoferlin (*MYOF*), named circMYOF, in PDAC [98]. CircMYOF was upregulated in PDAC tissues and cell lines, acted as a sponge of miR-4739, and regulated its target vascular endothelial growth factor A (*VEGFA*), leading to the activation of PI3K/AKT signaling and increased aerobic glycolysis (Warburg effect). The circMYOF/miR-4739/*VEGFA* axis facilitates PDAC progression and may serve as a prognostic biomarker and therapeutic target. A similar observation was made regarding the circ-membrane-bound O-acyltransferase domain containing two (circ-MBOAT2)/miR-433-3p/glutamic-oxaloacetic transaminase 1 (*GOT1*) axis [99]. In pancreatic cancer tissues and cells, circ-MBOAT2 and *GOT1* expression were significantly upregulated, while miR-433-3p expression was downregulated. Circ-MBOAT2 acted as a sponge of miR-433-3p, which was associated with *GOT1* and repressed cell proliferation, migration, invasion, and glutamine catabolism in pancreatic cancer.

**Table 3:** MiRNAs as potential targets for therapies in PDAC.

MiRNA	Expression	Validated Targets	Carcinogenic Effects	Reference
miR-21	Up	*PTEN*, *PDCD4*, *Spry2* MAPK/ERK and PI3K/AKT signaling pathways	Chemoresistance, activated cell migration and invasion	[91,92]
miR-27	Up	*BTG2* Wnt/β-catenin pathway	Enhanced angiogenesis	[93]
miR-194-5p	Up	*PD-L1*	Enhanced anti-tumor immunity	[28]
miR-29a	Up	*TTP* EMT	Pro-inflammatory, increased cell viability, invasion	[100]
miR-15a	Down	*WEE1*, *CHK1*, *Yap-1*, *BMI-1*	Induced cell cycle arrest and inhibition of cell proliferation	[94]
miR-145	Down	*MUC13*, *TGF-β* receptor, and *SMAD2*	Suppressed cell proliferation, migration, invasion	[95,96]
miR-34	Down	*Snail1*, *Notch1* EMT and Notch signaling	Induced apoptosis and inhibition of migration and invasion	[97]
miR-873	Down	*KRAS*	Suppressed proliferation	[101]
miR-4739	Down	*VEGFA* PI3K/AKT signaling, aerobic glycolysis	Inhibited cell growth and metastasis	[45]
miR-433	Down	*GOT1* glutamine catabolism	Repressed cell proliferation and metastasis and activated apoptosis	[99]

## 3. Challenges, Solutions, and Future Directions

While miRNAs from various sources show immense promise as biomarkers in pancreatic cancers, several challenges persist. The standardization of sample collection, isolation methods, and data analysis remains critical for these endeavors. Previous research, as reviewed in the preceding sections, has predominantly focused on exploring aberrant miRNA expression using microarray or sequencing profiling alongside bioinformatics analysis to identify key potential biomarkers. However, several issues were revealed.

### 3.1. Discrepancies in Reported Biomarkers

As evidenced in Table 1 and Table 2, different studies using distinct cohorts have reported divergent biomarkers, even based on similar statistical analysis. To further assess this issue, we conducted a comparative analysis using three datasets of tissue miRNA expression profiles and three datasets of serum miRNA expression profiles from TCGA and GEO, as summarized in Table 4.

Our differential expression analysis using the Limma package revealed several key observations, as follows.

- In tissue, there are 250, 93, and 83 differentially expressed (DE) miRNAs identified in each of the three datasets, respectively, with a fold change >2 and *p*-value < 0.05 (detailed lists in Supplementary Materials (Table S1)).
  ○ No single common DE miRNAs were found among all three datasets.
  ○ Two, nine, and twenty-two DE miRNAs were common among 2/3 datasets.
- In serum, 238, 122, and 203 DE miRNAs are identified in each of the three datasets, respectively, using less stringent criteria (Table S2).
  ○ Two common DE miRNAs were found among all three datasets, namely, miR-1246 and miR-1290.
  ○ Eleven, fourteen, and twenty-six DE miRNAs were common among 2/3 datasets.
- Tissue vs. serum groups: 117 miRNAs were differentially expressed in one or more datasets of each group (Table S3).
  ○ No single miRNA is common in all six datasets.
  ○ MiR-1246 is common in all serum datasets and 2/3 tissue datasets, while miR-205-5p is common in 2/3 of each group. However, the dysregulation trend is not consistent.

It is well noted that the discrepancies in methodology, patient cohorts, and clinical settings across studies likely contribute to these inconsistencies. The inherent heterogeneity of pancreatic cancer further complicates the identification of universal biomarkers, as tumors can exhibit diverse molecular subtypes, and patient responses to treatment can vary significantly. The inconsistency of the DE miRNAs could also suggest that the miRNAs’ role in pancreatic cancer may be more complex than initially thought, possibly influenced by various biological mechanisms and clinical factors. Thus, a single or small set of miRNAs may not adequately capture this heterogeneity, necessitating the exploration of composite biomarkers.

Moving forward, efforts should focus on the following.

1. Standardizing methodologies for exosome isolation, miRNA extraction, quantification, and data normalization across studies.
2. Exploring associated clinical metadata to understand how variables such as disease subtypes, stages, previous treatments, or comorbidities influence miRNA expression patterns.
3. Investigating composite biomarkers that combine miRNAs with clinical characteristics or other molecular markers such as differentiation 82 (CD82), CA 19–9, exosomal proteins (e.g., zinc transporter protein 4 (ZIP4) and Glypican-1 (GPC1)), and other types of non-coding RNAs for improved effectiveness in capturing the heterogeneity and complexity.
4. Integrating functional analysis to predict gene targets and associated pathways, enhancing the relevance of miRNAs to pancreatic cancer.

### 3.2. Discrepancies in Analytical Methods

Advancements in computational and statistical methods have enabled the analysis of large-scale miRNA expression data for cancer detection. Logistic regression, LASSO regression, and several machine learning models, such as Support Vector Machine and Decision Tree Classifier, have been applied to identify potential miRNA signatures [42,59,74]. However, given the complexity and heterogeneity of pancreatic cancer, the high dimensionality and noise inherent in miRNA expression data demand careful consideration.

One of the foremost challenges is selecting the most relevant miRNAs (features) from the vast pool. Feature selection techniques are critical for identifying informative miRNAs that discriminate between cancer and normal samples. However, striking a balance between identifying the most discriminatory miRNAs while avoiding overfitting and ensuring generalization is a delicate task. Developing robust feature selection algorithms that effectively filter out noise and irrelevant miRNAs while capturing critical ones contributing to the disease phenotype is imperative [102].

Another challenge arises from the need for more well-annotated and sufficiently large datasets. While machine learning algorithms, particularly deep learning models, thrive on big data to learn complex patterns effectively, obtaining large and well-curated miRNA expression datasets for pancreatic cancer remains challenging due to the rarity of the disease and associated difficulties in sample collection and data sharing. Limited data can lead to model overfitting and reduced generalizability.

Therefore, it is crucial to develop machine learning models that can effectively handle small datasets and extract meaningful information. These models must be designed to account for variability and be robust enough to accommodate the diverse miRNA expression patterns across different patient groups. Another challenge lies in interpreting the biological significance of selected miRNA biomarkers. Machine learning models often operate as ‘black boxes’, making it elusive to understand why specific miRNAs were chosen as biomarkers. Developing methods that predict biomarkers and provide insights into their functional roles and underlying biological mechanisms can enhance the credibility and clinical relevance of the identified biomarkers [54,98].

To illustrate a standard approach to identify miRNA signatures, we conducted a case study using a public dataset. This study involved the following.

- The utilization of example datasets from GSE59856, comprising serum miRNA expression data of 100 pancreatic cancer patients and 150 healthy controls.
- The use of Principal Component Analysis (PCA) for dimensionality reduction and visualization to understand the distribution of normal healthy and pancreatic cancer samples.
- The employment of the Limma package for differential gene expression analysis, aiding in the identification of top circulating miRNA biomarkers differentiating cancer patients from healthy controls.
- The construction of binary cancer classification models using several machine learning algorithms, including SVM, Decision Tree Classifier, Linear Discriminant Analysis (LDA), Quadratic Discriminant Analysis (QDA), and Gaussian Naive Bayes. The models’ performance was evaluated using k-fold cross-validation and standardized evaluation metrics such as accuracy, recall, precision, and F1 score.

As detailed in the Supplementary Materials (Table S4 and Figures S1 and S2), the distinct sample distributions between pancreatic cancer patients and healthy controls, as visualized through PCA, underscore the potential of these miRNAs to distinguish between these two groups. Regarding feature selection, SVM outperformed other models in the classification task and obtained the best model. After examining signatures of different sizes, a combinatory signature comprising miR-125a-3p, miR-6893-5p, miR-125b-1-3p, miR-6075, and miR-4294 was identified, demonstrating the best diagnostic accuracy of 97.59% and an AUC of 99.70%. These findings again highlight the potential of miRNAs as diagnostic biomarkers for pancreatic cancer.

In conclusion, identifying robust and efficient combinatory miRNA biomarkers in pancreatic cancer using feature selection and machine learning is a multifaceted challenge, which requires addressing feature selection, heterogeneity, dataset size, and biological interpretation issues. Addressing these challenges will advance the field towards more accurate diagnostic and prognostic tools, paving the way for personalized approaches to managing pancreatic cancer.

### 3.3. Alternative Approaches

While circulating exosomal miRNAs hold the promises, there are still challenges in handling exosome extraction, purification, and qualification in terms of their small RNA content, making it difficult to profile exosomal miRNA expression in pancreatic cancer tissues and biofluids. Studying the molecular properties of exosomal miRNAs may aid in overcoming these challenges and lead to discoveries in this field [103]. For instance, by performing motif analysis using MDS^2^ [104] among 880 exosomal miRNA sequences extracted from the ExoCarta database [105], we identified a few conserved sequence motifs for exosomal miRNAs (details in the Supplementary Materials (Table S5)). The motifs’ coverage, information content, and *p*-value were determined, revealing specific motifs highly enriched in miRNAs associated with human exosomes. These motifs may play a crucial role in packaging miRNAs into human exosomes, guided by RNA-binding transporter proteins [106]. Considering exosomal miRNA packaging may be a cell-specific process [103], future analysis can focus on exosomal miRNAs detected in specific pancreatic cancer tissue, cells, or biofluids. Understanding these sequence motifs could provide insights into exosomal miRNA packaging mechanisms and guide more target biomarker searches. MiRNAs associated with such motifs are more likely present in the exosomes, even in a low abundance in the early stage of the disease. However, further research is warranted to validate these findings and explore the mechanisms underlying these miRNAs’ roles in pancreatic cancer pathogenesis.

## 4. Conclusions

MiRNAs and exosomal miRNAs have emerged as promising biomarkers for pancreatic cancer diagnosis, prognosis, and targeted therapy. Their unique features, such as stability and accessibility through liquid biopsies, highlight their potential to transform the landscape of pancreatic cancer management. The results of prior research have provided compelling evidence for the potential of circulating miRNAs as non-invasion biomarkers for pancreatic cancer detection. Continued research and validation efforts are essential to unlock the full clinical potential of these biomarkers.

Despite the promising findings, several challenges must be addressed before miRNAs can be routinely used in clinical practice for pancreatic cancer management. Standardization of sample collection, RNA isolation, and miRNA profiling methods are essential to ensure reproducibility and comparability of the results between different studies. Additionally, large-scale prospective studies are needed to validate serum miRNAs’ clinical utility and incorporation into existing diagnostic and prognostic models. New paradigms to explore combinatory signatures by integrating miRNAs with other diagnostics modalities, including clinical information or mechanistic features, enable the identification of markers with enhanced sensitivity and specificity of pancreatic cancer detection, ultimately improving patient prognosis and survival rates. Moreover, specific miRNAs’ functional roles and regulatory mechanisms in pancreatic cancer pathogenesis need further exploration.

In conclusion, circulating miRNAs hold great promise as non-invasive biomarkers for pancreatic cancer, offering potential improvements in early detection, prognosis, and treatment monitoring. As research in this field advances, circulating miRNAs may become valuable tools in the fight against this deadly disease.

## Figures and Tables

**Table 1:** MiRNAs as diagnostic biomarkers in pancreatic cancer tissue, fluids, and exosomes.

MiRNA(s)	Expression	Sample	Population	Quantification and Analytic Methods	Ref.
miR-483-3p	Up	Tissue	107 PDAC patients and 22 controls	Microarray Mann–Whitney U test/Kruskal–Wallis test/ROC AUC (0.91)	[41]
		Serum	67 PDAC patients and 22 controls		
miR-1307	Up	Tissue	60 PDAC patients	High-throughput screening (HTS) Student’s *t*-test/ANOVA	[11]
miR-21-5p, miR-23a-3p, miR-31-5p, miR-34c-5p, miR-93-3p, miR-135b-3p, miR-155-5p, miR-196b-5p, miR-203, miR-205-5p, miR-210, miR-222-3p, miR-451, miR-622	Up	Tissue	165 PDAC, 59 AC, 6 DC, 21 DCBDC, and 39 CP patients, and 35 controls	RT-PCR Lasso-classifier	[42]
miR-122-5p, miR-130b-3p, miR-216b, miR-217, miR-375	Down				
miR-215-5p, miR-122-5p, miR-192-5p	Up	Tissue	27 PDAC and 23 CP patients, and 10 controls	sRNA sequencing Mann–Whitney test/AUC (0.720–0.988)	[67]
miR-30b-5p, miR-320b	Down	Serum	50 PDAC and 50 CP patients, and 25 Controls	RT-PCR ANOVA/AUC (0.720–0.988)	
mir-744-5p, mir-409-3p, mir-128-3p	Down	Serum	24 PC and 10 BTC patients, and 21 controls	sRNA sequencing edgeR/KNN	[68]
miR-22-3p, miR-642b-3p, miR-885-5p	Up	Plasma	35 PDAC patients and 15 Controls	qRT-PCR Chi-square test/Fisher exact test/Mann–Whitney/Kruskal–Wallis tests /ROC AUC (0.85–0.91)	[69]
miR-25	Up	Serum	80 PC patients and 91 controls	Mann–Whitney tests/Wilcoxon test	[10]
			303 PC patients and 600 controls	qRT-PCR ROC AUC (0.915)	[70]
			75 PDAC patients, 75 patients with benign lesions, and 100 controls	qRT-PCR Mann–Whitney test/ROC AUC (0.88)	[71]
miR-34a-5p, miR-130a-3p, miR-222–3p	Up	Plasma	Discovery: 58 PDAC patients and 30 controls Validation: 78 PDAC patients and 43 controls	Abcam Fireplex-Oncology Pane Benjamini and Hochberg/Logistic regression/ROC AUC (0.70–0.77)	[72]
miR-1246	Up	Blood/urine	41 PDAC patients and 30 controls	qRT-PCR Wilcoxon’s signed-rank test, Spearman’s rank correlation coefficient	[73]
miR-5100, miR-22-3p, miR-4486, let-7b-5p	Up	Serum	Discovery: 107 PC patients and 19 controls Validation 1: 25 PC and 81 ICC patients Validation 2: 11 PC, 8 ICC patients, and 8 controls	Microarray/qRT-PCR ROC AUC (0.98) SVM/accuracy (0.93)	[59]
miR-155-5p, miR-7154-5p, miR-661, miR-4703-5p	Down				
[mir-125a-3p, mir-5100 and mir-642b-3p] *	-	Serum	Discovery: 342 PC patients and 329 controls Validation: 81 PC patients and 70 control	Microarray datasets from GEO, normalized by SAM Logistic regression/ROC AUC (0.95)	[74]
[miR-132-3p, miR-30c-5p, miR-24-3p, miR-23a-3p] *	Up	Tissue	Discovery: 200 PC patients and 100 controls	Microarray/RT-PCR Limma/*t*-test/Chi-squared test/ROC AUC (0.971)	[75]
		Serum	Training: 285 PDAC and 45 CP patients, 197 controls, and 108 patients with other pancreatic diseases Validation: 286 PDAC and 45 CP patients, 198 controls, and 109 patients with other pancreatic diseases	RT-PCR Student’s *t*-test/ANOVA/multiple linear regression	
2′-O-methylated (2′OMe) [miR-28-3p, miR-143-3p, miR-151a-3p] *	Up	Plasma	10 PDAC patients and 10 controls	sRNA sequencing Multivariate analysis/ROC AUC (0.928)	[76]
[miR-125a-3p, miR-4530, miR-92a-2-5p]	Up	Plasma	77 PC patients and 65 controls	qRT-PCR Student’s *t*-test/ANOVA/Chi-square test ROC AUC (0.86)	[77]
miR-1246, miR-196a	Up	Plasma exosome	15 PDAC patients and 15 controls	qRT-PCR Student’s *t*-test/Wilcoxon/Mann–Whitney/ROC AUC (0.71–0.81)	[78]
mi-191, miR-21, miR-451a	Up	Serum exosomes	32 PC and 29 IPMN patients, and 22 controls	qRT-PCR Mann–Whitney test/ROC AUC (0.76–0.83)	[64]
miR-21, miR-30c, miR-106b, miR-20a, miR-181a, miR-10b	Up	Serum exosome	29 PDAC and 11 CP patients, and 6 controls	qRT-PCR ANOVA	[52]
miR-483, miR-122	Down				
miR-18a, miR-106a	Up	Plasma EV	20 PDAC patients and 20 controls	sRNA sequencing/RT-PCR ANOVA/Student’s *t*-test	[79]
miR-664a-3p	Up	Plasma EV	58 PDAC, 12 CP, and 12 BPT patients, and 20 controls	Student’s *t*-test/FDR/LASSO regression, RF, and SVM-RFE	[80]
miR-3940-5p, miR-8069	-	Urine exosome	43 PDAC and12 CP patients, and 25 controls	PCR Fisher exact test/Wilcoxon rank-sum test	[81]
[miR-1246, miR-4644] *	Up	Saliva exosome	12 PTC patients and 13 controls	qRT-PCR ROC AUC (0.833)	[82]
miR-20a	Up	Duodenal fluid EVs	27 PDAC patients and 7 controls	qRT-PCR Wilcoxon signed-rank test/ROC AUC (0.88)	[57]
miR-21, miR-155 (with pancreatic juice cytology)	Up	Pancreatic juice exosome	27 PDAC patients and 8 CP patients	qRT-PCR Wilcoxon signed-rank test/ROC AUC (0.89–0.90) Accuracy (91%)	[60]
miR-21-5p (with human satellite II RNA)	Up	Serum	Discovery: 30 PDAC patients and 30 controls Validation: 35 PDAC patients and 40 controls	Microarray/PCR Welch’s *t*-test/Fisher’s exact test/ROC AUC (0.90)	[56]

* Signature miRNAs enclosed in a square parenthesis are used as a combinatory panel. Abbreviations: PDAC (pancreatic ductal adenocarcinoma), AC (duodenal cancers), DC (duodenal cancers), DCBDC (distal common bile duct carcinoma), CP (chronic pancreatitis), BTC (biliary tract cancer), PTC (pancreaticobiliary tract cancer), IPMN (intraductal papillary mucinous neoplasm), ICC (intrahepatic cholangiocarcinoma), EV (extracellular vesicle), BPT (benign pancreatic tumor), KNN (k-nearest neighbor), SAM (significance analysis of microarrays), SVM (support vector machine), RFE (recursive feature elimination), RF (random forest).

**Table 2:** MiRNAs as prognostic biomarkers in pancreatic cancer tissue, fluids, and exosomes.

MiRNA(s)	Expression *	Sample	Population	Quantification and Analytic Methods	Ref.
miR-451a	Up/Positively correlated	Plasma	Discovery: 6 PDAC patients (and 3 controls) Validation: 50 PDAC	Microarray Cox proportional hazards regression	[47]
miR-221-3p	Up/Positively correlated	Plasma	87 PC patients (and 48 controls)	qRT-PCR Chi-square test/Fisher’s exact probability test/ROC AUC	[29]
miR-21	Up/Positively correlated	Serum exosome	32 PC patients (and 22 controls)	sRNA sequencing/qRT-PCR Kaplan–Meier with a log-rank test and Cox proportional-hazards regression model	[64]
miR-370-3p	-/Positively correlated	Plasma	Discovery: 7 PDAC patients Validation: 113 PDAC patients	sRNA sequencing/qRT-PCR multivariate analysis	[84]
miR-31-5p, miR-205-5p	Up/Positively correlated	Tissue	Discovery: 58 PDAC patients Validation: 179 PDAC patients (TCGA)	PCR array/RT-qPCR Student’s *t*-test/ROC	[85]
miR-200b	-/Positively correlated	Cyst fluid EV	Discovery: 6 M-PCN and 7 non-M-PCN patients Validation: 24 M-PCN and 30 non-M-PCN patients	PCR Mann–Whitney U-test/Chi-square test	[86]
miRNA-132	Down/Negatively correlated	Tissue	Discovery: 50 PDAC patients (and 50 controls) Validation: 179 PDAC patients (TCGA)	qRT-PCR Chi-square test/Fisher exact test/Cox proportional hazards regression	[30]
miR-7	Down/Negatively correlated	Tissue	Discovery: 8 PDAC patients (and 3 controls) Validation: 179 PDAC patients (TCGA)	qRT-PCR microarray Student’s *t*-test/Chi-square test/Kaplan–Meier/Spearman’s rank correlation	[32]
miR-26a-5p	Down/Negatively correlated	Tissue	96 PDAC patients	qRT-PCR Student’s *t*-test/Chi-square test/Kaplan–Meier	[87]
miR-424, miR-3613, miR-4772, miR-126	-/-	Tissue	179 PDAC patients (TCGA) 45 PDAC patients (GSE28735)	Public sequencing and microarray data Cox proportional hazards regression	[53]
let-7g, miR-29a-5p, miR-34a-5p, miR-125a-3p, miR-146a-5p, miR-187, miR-205-5p, miR-212-3p, miR-222-5p, miR-450b-5p	-/-	Tissue	103 PDAC patients and 54 A-AC	PCR logistic regression/Kaplan–Meier/Cox proportional hazards regression	[42]
miR-574-5p, miR-1244, miR-145, miR-328, miR-26b, and miR-4321	-/-	Tissue	178 PDAC patients (TCGA)	Public sequencing data Pearson Correlation Analysis/Cox proportional hazards regression	[54]
miR-20a-3p (with circ-0005105/COL11A1)	Down/-	Tissue	170 PC patients	sRNA sequencing DESeq2/ANOVA	[88]
miR-6820-3p (with circ_0007367)	Down/-	Tissue	128 PDAC patients	qRT-PCR Kaplan–Meier method and log-rank test/Pearson correlation coefficient	[89]

* Expression (in patients vs. control/correlation with poor prognosis, e.g., poor outcomes, recurrences, or advanced disease stages). Abbreviations: M-PCN (mucinous pancreatic cystic neoplasms), A-AC (ampullary adenocarcinoma).

**Table 4:** Overview of major research discrepancies, challenges, and solutions illustrated by the case study.

Category	Reported Biomarkers	Analytical Methods
Dataset and analysis	Tissue (TCGA-PAAD, GSE24279, GSE119974) and serum (GSE59856, GSE85589, GSE109319) Differential expression (DE) analysis by two different methods	Serum GSE59856 dataset for diagnostic marker identification PCA used for sample distribution visualization, Limma for DE analysis, and different machine learning methods for signature identification
Key observations	Divergent DE miRNAs across different datasets Few common DE miRNAs between tissue and serum groups	Best signatures vary with different methods, exhibiting varying levels of classification power
Underlying Challenges	Discrepancies introduced by methodology, patient cohort, and cancer heterogeneity	Feature selection complexity and dataset limitations Balancing discriminatory power and generalizability Biological interpretation of miRNA biomarkers
Recommendation	Standardization of quantification and analytical methodologies Exploration of clinical metadata Investigation of composite biomarkers Integration of functional analysis	Employment of robust feature selection Acquisition of large and well-curated datasets Design of models accommodating small datasets and variability

## Supplementary Materials

The following supporting information can be downloaded at: https://www.mdpi.com/article/10.3390/ijms25073914/s1.
